# Autoimmune Polyglandular Syndrome Type 3c with Ectodermal Dysplasia, Immune Deficiency and Hemolytic-Uremic Syndrome

**DOI:** 10.4274/Jcrpe.1128

**Published:** 2014-03-05

**Authors:** Mithat Büyükçelik, Mehmet Keskin, Özlem Keskin, Ali Bay, Beltinge Demircioğlu Kılıç, Yılmaz Kor, M. Arda Kılınç, Ayşe Balat

**Affiliations:** 1 Gaziantep University Faculty of Medicine, Department of Pediatric Nephrology, Gaziantep, Turkey; 2 Gaziantep University Faculty of Medicine, Department of Pediatric Endocrinology and Metabolism, Gaziantep, Turkey; 3 Gaziantep University Faculty of Medicine, Department of Pediatric Allergy and Immunology, Gaziantep, Turkey; 4 Gaziantep University Faculty of Medicine, Department of Pediatric Hematology, Gaziantep, Turkey; 5 Gaziantep University Faculty of Medicine, Department of Pediatrics, Gaziantep, Turkey

**Keywords:** Polyglandular syndrome, ectodermal dysplasia, immune deficiency, Hemolytic uremic syndrome

## Abstract

Autoimmune polyglandular syndrome (APS) is a disorder which is associated with multiple endocrine gland insufficiency and also with non-endocrine manifestations. The pathophysiology of APS is poorly understood, but the hallmark evidence of APS is development of autoantibodies against multiple endocrine and non-endocrine organs. These autoantibodies are responsible for the dysfunction of the affected organs and sometimes may also cause non-endocrine organ dysfunction. The hemolytic-uremic syndrome (HUS) is a serious and life-threatening disease which develops due to many etiological factors including autoimmune disorders. Here, we present an unusual case of APS. Ectodermal dysplasia with immune deficiency and HUS occurred concomitantly in the same patient with APS type 3c. Once the autoantibody generation was initiated in the human body, development of multiple disorders due to organ dysfunction and also autoantibody-related diseases may have occurred.

## INTRODUCTION

Autoimmune polyglandular syndrome (APS) refers to a group of autoimmune disorders characterized by dysfunction in two or more endocrine glands and is associated with circulating organ-specific antibodies directed against the involved glands (1,2,3). The most frequently encountered APSs are adrenal insufficiency, autoimmune thyroid disease, insulin-dependent diabetes mellitus, hypoparathyroidism and premature gonadal failure. The underlying abnormality responsible for polyglandular autoimmunity is poorly understood, but most likely a defect in T suppressor cell function may have a role in the pathophysiology of APS. There is evidence that aberrant expression of HLA DR antigens may also have an important role in the pathogenesis of these disorders (3).

Ectodermal dysplasia with immune deficiency is an X-linked immunological and developmental disorder caused by mutations in the gene encoding nuclear factor κB (**NF-κB**) essential modulator (NEMO) (4). **NF-κB** is a master transcriptional regulator critical for ectodermal development and adaptive immune function (4).

Hemolytic-uremic syndrome (HUS) is characterized by the triad of microangiopathic hemolytic anemia, thrombocytopenia and acute renal failure. The pathogenesis of HUS is mainly related to a mutation in complement regulatory protein factor H, factor I or factor B, or to the presence of autoantibodies against factor H (5,6,7,8). HUS has been also associated with various nonenteric infections, viruses, drugs, malignancies, transplantation, pregnancy and other underlying antibody-mediated autoimmune disorders such as scleroderma, systemic lupus erythematosus (SLE) and antiphospholipid syndrome (6,7).

Herein, we describe a patient with APS type 3c with ectodermal dysplasia, immune deficiency and HUS. To our knowledge, this is the first case to be reported in the relevant literature.

## CASE REPORT

A ten-year-old girl was admitted to our hospital with complaints of cough, fever, vomiting, weakness and lack of appetite. She also suffered from alopecia and persistent/recurring respiratory infections. Both parents were reported to be healthy and they were consanguineous (first cousins). Their four children except for our patient were healthy.

**Figure 1:**
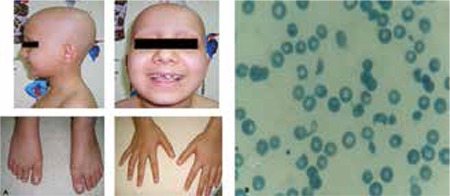
a) total alopecia and nail pitting, b) blood smear showing the hemolysis

Physical examination of the patient revealed normal vital signs. The percentiles of her weight and height were between the 3^rd^ and 10^th^ percentiles. She had pale skin, total alopecia and pitting in the nails of her hands and feet (Figure 1a). A crackling sound was heard during inspiration. Her puberty was stage I according to Tanner stages.

Her hematology results revealed a hemoglobin level of 7.0 g/dL, hematocrit 19.9%, white blood cells 13200/mm^3^, platelets 35000/mm^3^, MCV 84fL and reticulocytes of 8.14%, as well as negative direct Coombs test result. Blood smear showed schistocytes (Figure 1b). Her biochemical investigation revealed: urea 142 mg/dL, creatinine 4.61 mg/dL, AST 97 U/L, ALT 79 U/L, LDH 2218 U/L, total bilirubin 1.5 mg/dL, direct bilirubin 0.55 mg/dL, sodium 134 mmol/L, potassium 5.9 mmol/L, chloride 109 mmol/L, calcium 8.7 mg/dL, phosphorus 4.0 mg/dL, uric acid 8.0 mg/dL, ph 7.44, HCO_3_- 21 mmol/L and glucose 76 mg/dL. The urinalysis showed the following: pH 7.5, density 1010, protein (++), erythrocytes (+++), leucocytes (-) and dysmorphic erythrocytes in the urinary sediment.

Based on these biochemical and urinary abnormalities, the patient was diagnosed as HUS. After seven plasma exchange sessions, the hemolysis finally stopped and the laboratory abnormalities and renal function reverted to normal. The respiratory infection was treated with appropriate antibiotic regimens.

In the hormonal evaluation, free thyroxine (fT4) was 1.11 ng/dL (0.8-2.2), thyroid-stimulating hormone (TSH) 3.028 mU/mL (0.6-5.5), anti-peroxidase (anti-TPO) antibody 62 IU/ mL (<20), anti-thyroglobulin (anti-TG) antibody 65 IU/mL (<4.0), cortisol 25.5 mg/dL (3-21), adrenocorticotropic hormone (ACTH) 28.1 pg/mL (10-60), estradiol 14 ng/dL (0.2.0), follicle-stimulating hormone (FSH) 0.32 mIU/mL, luteinizing hormone (LH) 0.07 mIU/mL (0.02-0.3), anti-pancreatic islets antibody 1/100 (<1/10), parathyroid hormone (PTH) 65.9 ng/mL (10-65), renin 72 ng/L (5-35), aldosterone 248 pg/mL (20-300), vitamin B12 456 pg/mL (150-883) and folic acid 4.2 ng/mL (3.1-20.5) before plasma exchange therapy. Although thyroid ultrasonography was normal and the patient was not on Na-L-T4 treatment, both anti-TPO antibody and anti-TG antibody tests were positive. These findings suggested early diagnosed autoimmune thyroiditis. Cortisol response to ACTH stimulation test (1 μg ACTH) was as follows: initial 1 mg/dL, 30^th^ minute 8.1 mg/dL, 60^th^ minute 11.2 mcg/dL, 90^th^ minute 14.1 mg/dL, and 120^th^ 14.7 mg/dL. The cut-off level of this test was 14 mg/dL (9). These results were interpreted as a normal response to ACTH stimulation test and indicated that there was no adrenal insufficiency. In immunological evaluation, IgA was 24 mg/dL (29-384), IgM 24 mg/dL (50-278), IgG 464 mg/dL (625-1579), IgE 464 IU/mL (0-170), C3 90 mg/dL (90-180), C4 26 mg/dL (10-40), anti-nuclear antibody (ANA) (-), anti-double strain DNA antibody (-), p-ANCA (-), c-ANCA (-) and anti-HBs (-) before plasma exchange therapy. Lymphocyte subsets were CD3 78% (55-78), CD19 2% (10-31), CD4 39% (27-53) and CD8 51% (19-34). The complement factor H gene region was proliferated by PCR method. Sequences which have single nucleotide polymorphism on complement factor H have been determined. CT homozygote polymorphism for His402Tyr was detected. This factor is considered to be a risk factor for development of HUS (10). In radiological evaluation, pulmonary X-ray showed bilateral diffuse infiltration and abdominal ultrasonography was normal except for hepatomegaly and grade I increase in renal echogenicity.

## DISCUSSION

APS are a group of disorders which include multiple endocrine gland insufficiency associated with autoimmune disease. Neufeld and Blizzard in 1980 suggested a classification of APS based on clinical criteria and described four main types (3): APS-1- chronic candidiasis, chronic hypoparathyroidism, Addison’s disease (at least two present); APS-2- Addison’s disease (always present) + autoimmune thyroid disease and/or type 1 diabetes mellitus; APS-3- autoimmune thyroid diseases associated with other autoimmune diseases (excluding Addison’s disease and/or hypoparathyroidism); APS-4- combinations not included in the previous groups. APS-1 is a very rare syndrome in young subjects and is related with different mutations of auto immune regulator gene on chromosome 21. APS-2 is a rare syndrome in adult females and is associated to a genetic pattern of HLA DR3/DR4. APS-3 was defined as the association between a clinical entity of autoimmune thyroid diseases and another autoimmune disease such as type 1 diabetes mellitus (Type 3a), chronic atrophic gastritis, pernicious anemia (Type 3b), vitiligo, alopecia, myasthenia gravis (Type 3c), other unspecified diseases (Type 3d). In the following years, APS-3 appeared to be more complicated than initially reported by Neufeld and reviewed according to the clinical, genetic and immunological aspects of this syndrome (Table 1).

HUS is characterized by the triad of microangiopathic hemolytic anemia, thrombocytopenia and acute renal failure. Two types of HUS have been described: one of these is D (+) or shiga-toxin related, is also the more common form seen in childhood, the other is D (-) or named atypical HUS (5,6,7,8). Atypical HUS is responsible for 10-15 percent of all HUS cases encountered in children and is associated with various non-enteric infections, drugs, malignancies, transplantation and other underlying autoimmune diseases such as scleroderma, anti-phospholipid syndrome and SLE. Although thrombotic thrombocytopenic purpura (TTP) is related to the deficiency of metalloprotease ADAMTS 13 which is involved in the regulation of von Willebrand factor, both HUS and TTP share the same findings (5,6,7,8). Therefore, HUS and TTP fall into the broader category of thrombotic microangiopathies. Atypical HUS mainly develops due to mutations in the genes encoding the complement proteins including C3, factors H, B, I and CD46 (membrane cofactor protein, MCP). These mutations are responsible for approximately 50 percent of cases of atypical HUS and may result in dysregulation of the complement system associated with excessive complement activation that causes endothelial damage (6). In the present case, we detected CT homozygote polymorphism for His402Tyr amino acid in complement factor H gene region. The patient did not have adrenal cortex failure, but she had thyroid disease with anti-thyroid antibodies and total alopecia. These findings point to the presence of APS-3 as a cause of multiple endocrine organ failure in our case. There was also evidence of atypical HUS in our patient, including hemolytic anemia and thrombocytopenia. These clinical findings were as in Type 3c (Table 1). Although we have not demonstrated any autoantibody against factor H as a cause of HUS, it may probably arise as a part of triggered autoimmunity responsible for the development of multi-endocrine organ failure like SLE in this patient. Therefore, following the plasma exchange therapy, hematological abnormalities related to HUS recede and renal functions return to normal. In our patient, following plasma exchange therapy, HUS has not recurred and glomerular filtration rate remained within normal limits without any therapy for one year.

In the pathophysiology of APS, there is some degree of genetic susceptibility in these individuals and a subsequent exposure to the autoimmune trigger which could be an environmental or intrinsic factor. After the antigenic trigger, a subclinical phase of active production of organ-specific autoantibodies takes place. In our patient, there were recurrent infections and low levels of IgA, IgM, IgG and CD3 as in primary immune deficiency disorders with B cell defects. Genetic mutations in the NEMO may be responsible for this immune deficiency because the patient has also ectodermal dysplasia (4). Autoimmunity is frequently observed in patients with primary immune deficiency disorders (11,12). Many immune deficiency syndromes, mainly humoral defects, are associated with autoimmune disorders (11,12). Following this stage, autoimmune activity causes progressive glandular destruction by the inflammatory infiltrate mainly composed of lymphocytes, macrophages, natural killer cells and plasma cells in the respective organ and all this activity leads to the subsequent development of overt clinical disease.

In conclusion, the findings in the present patient show that, provided there is a genetic predisposition to develop an abnormal immune reaction, autoimmune response may be initiated by many intrinsic or extrinsic factors and multiple organ failure may develop depending on this immune response. Thus, as a part of the different reactions, various autoantibodies directed to endocrine and/or non-endocrine organs and also other proteins develop in the human body, as in HUS. We believe this patient with APS type 3c who presented with ectodermal dysplasia, immune deficiency and HUS occurring concomitantly, represents the first example of this unique combination.

## Figures and Tables

**Table 1:**
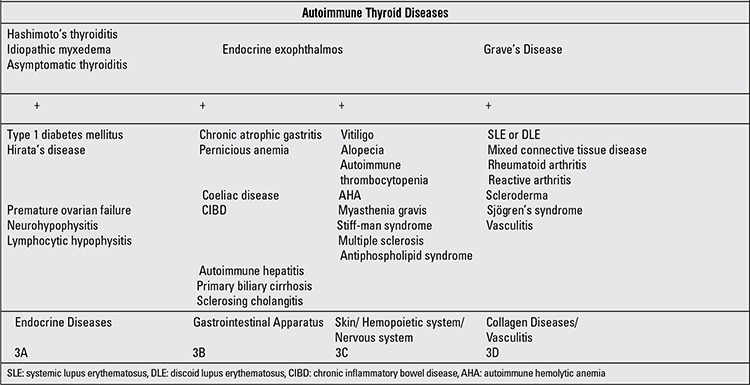
Characteristics of autoimmune polyglandular syndrome (APS) type 3 (modified from Betterle, 2003) ([ref:1]1[/ref])
